# Ginsenoside Re: Its chemistry, metabolism and pharmacokinetics

**DOI:** 10.1186/1749-8546-7-2

**Published:** 2012-02-07

**Authors:** Dacheng Peng, Huashan Wang, Chenling Qu, Laihua Xie, Sheila M Wicks, Jingtian Xie

**Affiliations:** 1 The Ben May Department for Cancer Research, Pritzker School of Medicine, The University of Chicago, Chicago, IL 60637, USA; 2 Section of Emergency Medicine, Pritzker School of Medicine, The University of Chicago, Chicago, IL 60637, USA; 3 College of Grain Oil and Food Science, Henan University of Technology, 140 Songshan South Road, Zhengzhou 450052, China; 4 Department of Cell Biol & Mol Med, UMDNJ-New Jersey Medical School, 185 South Orange Ave. MSB C609, Newark, NJ 07103, USA; 5 Department of Biological Sciences, City Colleges of Chicago, 1900 W. Van Buren Street, Chicago, IL 50512, USA; 6 University of Illinois at Chicago, 909 S. Wolcott Ave. Chicago, IL 60612, USA; 7 Shangqiu Medical College, 486 W. Beihei Street, Shangqiu, Henan 476000, China

## Abstract

Ginsenosides, the bioactive components of ginseng, can be divided into two major groups, namely 20(S)-protopanaxatriol (e.g. Re, Rg_1_, Rg_2_, and Rb_3_) and 20(S)-protopanaxadiol (e.g. Rb_1_, Rb_2_, Rc, and Rd). Biological and environmental factors may affect the content of ginsenosides in different parts of ginseng plant. Evidence from pharmacokinetic and metabolic studies of Re demonstrated that (1) the absorption of Re is fast in gastrointestinal tract; (2) Re may be metabolized mainly to Rh_1 _and F_1 _by intestinal microflora before absorption into blood; and (3) Re is quickly cleared from the body.

## Background

Ginseng is a key herb in Chinese medicine, and has a wide range of therapeutic and pharmacological uses [1-3]. *Panax ginseng *is a slow growing perennial herb of the Araliaceae family usually cultivated in China, Japan, Korea and Russia, as well as in the United States and Canada. Ginseng root has been used as an oriental folk medicine for several thousand years [2,4]. It is a highly valued medicinal plant in the Far East that and also popular in the West in the past 20 years [2,4-7].

A number of studies suggest that both *Panax ginseng *C.A. Meyer (also known as Asian ginseng, Chinese ginseng or Korea ginseng) and *Panax quinquefolius *(also known as American ginseng) have multiple components and pharmacological functions [7-14]. Among the complex constituents of ginseng, ginsenosides (also known as ginseng saponins or triterpene saponins) are the major components responsible for biochemical and pharmacological actions of ginseng [9,15-17]. With the development of modern technology, more than 150 naturally occurring ginsenosides have been isolated from *Panax *species [18]. About 40 ginsenosides have been identified from the root of *Panax ginseng *[1,19-22].

**Figure 1:**
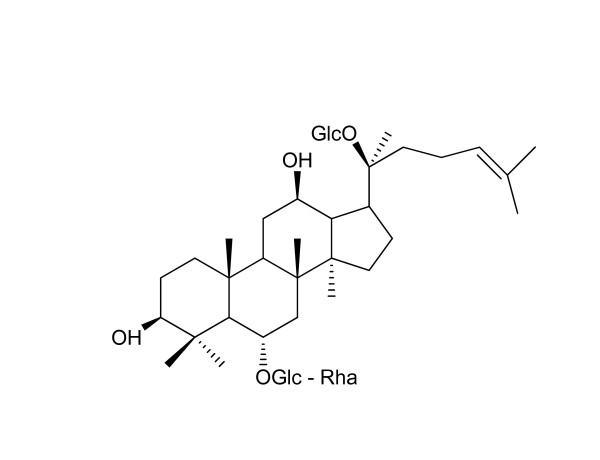
**Chemical structure of ginsenoside Re (MW = 946)**.

In order to explore the pharmacological actions, mechanisms and clinical applications of ginseng, some researchers focused on purified individual ginsenosides rather than whole ginseng extracts [1,23]. Individual ginsenosides may have different characteristics in chemistry, metabolism, and pharmacokinetics. Ginsenoside Re (Re) belongs to 20(S)-protopanaxatriol group (Figure 1), and is a major ginsenoside in ginseng [7,10,16,22,24-27]. Literature shows that Re exhibits multiple pharmacological activities *via *different mechanisms [12,16,28]. For example, in cardiovascular system, Re possesses negative effects on cardiac contractility and autorhythmicity, anti-arrhythmic and anti-ischemic effects, angiogenic regeneration activities and cardiac electrophysiological functions [28]. Xie *et al*. and Li *et al*. [13,29-32] found that the quantity of Re in ginseng leaf and berry is much higher than in ginseng main root and suggested that ginseng leaf-stem could be a valuable source for Re. There have been other new findings in recent years. This article provides an overview of the recent advances in chemistry, metabolism and pharmacokinetics of Re.

## Chemistry and content

### Chemical structure of Re

Rg_1_, Rc, Rd, Re, Rb_1_, Rb_2 _and Rb_0 _are the main ginsenosides in quantity [1,33]. The top six major ginsenosides (Rb_1_, Re, Rd, Rc, Rg_1 _and Rb_3_) make up over 70% of total ginsenoside content in *P. quinquefolius *[34,35].

**Figure 2:**
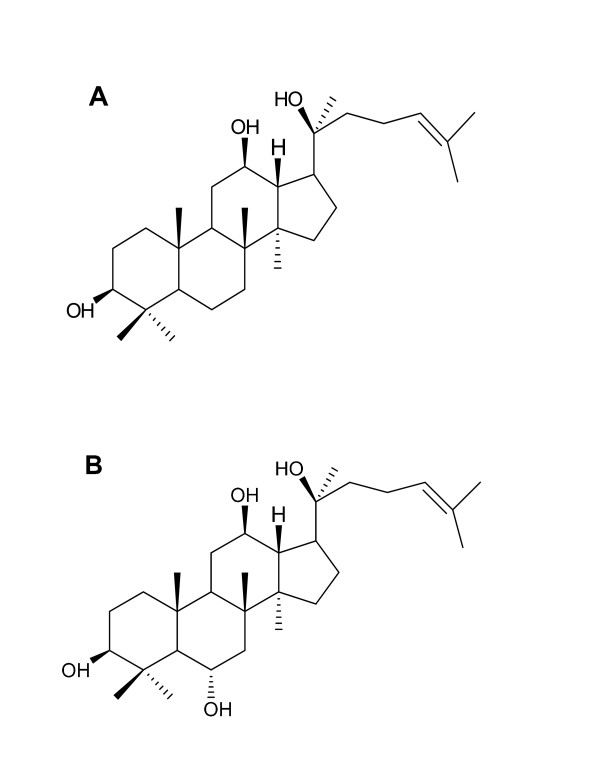
**Chemical structures of protopanaxadiol (A) and protopanaxatriol (B)**.

Ginsenosides are the glycosides that contain aglycone with dammarane (except Ro). Ginsenosides (Figure 2) are generally divided into two groups, namely the protopanaxadiol (PPD) and protopanaxatriol (PPT) ginsenoside groups. The sugar moities in the PPD group including Rb_1_, Rb_2_, Rc, Rd, Rg_3 _and Rh_3_, attach to the 3-position of dammarane-type triterpine, whereas the sugar moities in the PPT group including Re, Rf, Rg_1_, Rg_2 _and Rh_1_, attach to the 6-position of dammarane-type triterpine. A ginsenoside possesses a rigid four *trans*-ring steroid skeleton with a modified side chain at C-20 [1,17,36-39]. The chemical structures of ginsenosides are different from each other in the number, linkage position and type of sugar [17,39]. During extraction, sugar moieties of ginsenosides may be cleaved by acid hydrolysis or endogenous glycosidases to give corresponding aglycones [25,36]. PPD and PPT may rearrange into panaxadiol and panaxatriol, respectively, to provide artificial ginsenoside products. Kang *et al*. [40] showed that PPD and PPT ginsenoside groups had different bioactivities, even opposite effects. Recently, Zhu *et al*. [41] found six new PPT-type ginsenosides extracted from the *P. ginseng *root, named Re_1 _to Re_6 _(compounds 1-6) respectively, along with ten other known PPT ginsenosides.

### Content of Re in ginseng

The biological and environmental factors that may affect the quantity and quality of ginsenosides in ginseng [14,35] include the species, age, part of the plant, season of harvest, method of cultivation, and means of preservation. For example, the content of Re, Rg_1 _and Rd is higher in the wild *P. ginseng *roots than in the cultivated ginseng roots, while the content of Rc, Rb_2 _and Rb_1 _is lower in the wild *P. ginseng *roots than in the cultivated ones. These differences in content of ginsenosides might affect their biological and pharmacological properties. Root ginsenoside content depends on the age of ginseng plant. For example, the plants younger than four years of age are considered unsuitable for harvest due to their low ginsenoside content [35,42-44]. Lim *et al*. [35] determined the genotypes and environmental factors affecting the ginsenoside content among eight wild populations of *P. quinquefolius*. The influence of genotypes and environment on ginsenoside content varies among different types of ginsenosides. Specifically, the Re content varies with populations but not locations, whereas Rb_1_, Rc and Rb_2 _only varies with locations, and Rg_1 _and Rd varies with both. Ginsenoside levels are decreased, while ginseng growth is increased, at an intensively managed garden location. The content and composition of ginsenosides vary with other environmental conditions such as the type of soil, temperature, light intensity and water content [45].

Using high pressure microwave-assisted extraction (HPMAE) and high-performance liquid chromatography (HPLC) coupled with evaporative light scattering detection (ELSD), *i.e*. HPMAE HPLC-ELSD, Qu *et al*. [46] studied the effects of different parts and age of *P. quinquefolius *on the content of 12 ginsenosides, namely Rg_1_, Re, Rf, Rg_2_, Rh_1_, Rb_1_, Rc, Rb_2_, Rb_3_, Rd, Rh_2 _and F_11_. The study ranked the parts of five-years-old *P. quinquefolius *in terms of total content of these 12 ginsenosides in a descending order: leaf, root-hair, rhizome, main root and stem, suggesting that the leaf could be a better source for ginsenosides, as compared with other parts of ginseng plant. It also found that in ginseng roots, the content of Re and Rb_1_, the major ginsenosides, increase with age of the plant.

In a comparative study on the quality of Tongrentang Red Ginseng and Korean Red Ginseng, Wu *et al *[47] found that the content of Re, Rg_1 _and Rb_1 _in the Tongrentang Red Ginseng is less than the content in the Korean Red Ginseng.

Another extensive study [48] performed a quantitative analysis of Re, Rb_1 _and Rg_1 _in *P. quinquefolius *berry and flower sampled in various months throughout the year, by enzyme-linked immunosorbent assay (ELISA). The *P. quinquefolius *flower had higher content of Re, Rb_1 _and Rg_1 _and the lowest content of Re in the berries harvested in September [48]. To analyze the Re content in *P. quinquefolius *berry pulp extracts, Morinaga *et al*. [49] performed a new Eastern blot technique with anti-Re monoclonal antibody, and confirmed that the content of Re varies from part to part in the plant.

Lee *et al*. [50] reported the variations in the ginsenoside profiles of ginseng landraces in Korea. They found that the *P. ginseng *wild population exhibits three types of ginsenoside profiles affected by genetic and environmental factors.

## Metabolism and pharmacokinetics

Re has recently been studied extensively [12,13,30]; however, little is known about the metabolic and pharmacokinetic profiles.

### Absorption

After oral administration, Re is in contact with the gastriointestinal fluids containing gastric acids and gastric enzymes, intestinal enzymes, and colonic bacteria [51,52]. Li *et al*. [23,53] studied the pharmacokinetic parameters and absolute bioavailability of Re, R_1_, Rg_1_, Rd, and Rb_1 _after oral or intravenous administration of total notoginsenosides. Main pharmacokinetic parameters of these constituents were determined by Drug and Statistics (DAS) for Windows pharmacokinetics software. The results showed that Re, R_1_, Rg_1_, Rd and Rb_1 _reached peak concentration in plasma within about 45 minutes after oral administration of total panax notoginsenoside (TPNG) powder in rats, suggesting a rapid absorption of ginsenosides in gastrointestinal tract. The absolute bioavailability of Re was 7.06% [53]. To confirm the rapid absorption finding, Joo *et al*. [27] conducted a pharmacokinetic study using ICR mice and ultra performance liquid chromatography-electrospray ionization-mass spectrometry (UPLC-ESI-MS) analytical method. This pharmacokinetic study [27] revealed that the time to reach the peak plasma concentration after oral administration was 0.4 ± 0.2 hour. The data also showed that the oral bioavailability was 0.19-0.28%. Qi *et al*. [54] found that the oral bioavailability of PPD ginsenosides (Ra_3_, Rb_1_, Rd, Rg_3 _and Rh_2_) and PPT ginsenosides (Rg_1_, Re, Rh_1_, and R_1_) was less than 5% and PPT ginsenosides had better bioavailability, possibly due to the faster degradation of PPD ginsenosides.

### Metabolism and biotransformation

**Figure 3:**
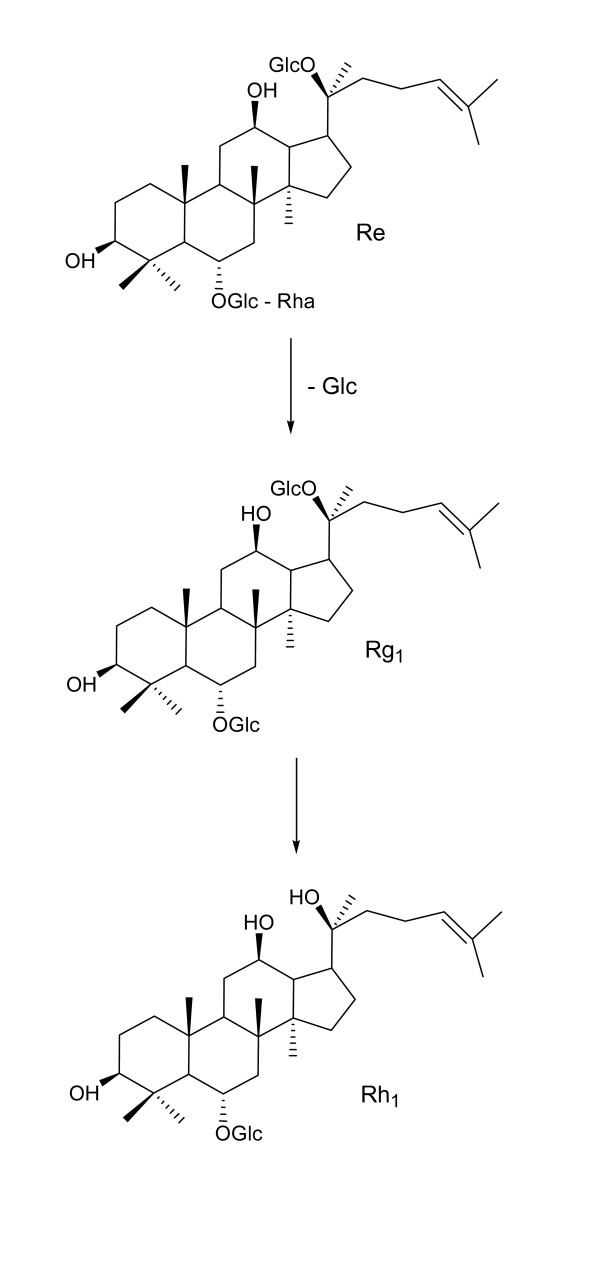
**Proposed possible metabolism pathway of G-Re by stomach acidity and large intestine bacteria**.

Han *et al*. [55] showed that PPT ginsenosides are hydrolyzable to Rh_1 _under mild acidic conditions. Tabaw *et al*. [56] found that two degradation products of the PPT ginsenosides, Rh_1 _and F_1 _could reach the systemic circulation in humans in addition to compound-K resulting from the stepwise deglycosylation of PPD ginsenosides. Bae *et al*. [57] further confirmed that the PPT (Re and Rg_1_) could be metabolized mainly to Rh_1 _and G-F1 in the gastrointestinal tract by intestinal microflora, before absorption into the blood. Chi and Ji *et al*. [58] tested the biotransformation of Re and Rb_1 _by cell extracts from various food-grade edible microorganisms. As shown in Figure 3, Re was transformed into Rh_1 _*via *Rg_2 _by *Bif. sp*. Int57 and *Bif. sp*. SJ32; *A. niger *transformed Re into Rh_1 _*via *Rg_1_; *A. usamii *transformed Re into Rg_2_. However, Rb_1 _was transformed into compound-K and Rh_1 _by different pathways [58].

Metabolic research of Re in animals was also reported [59]. Six SD rats were used and divided into three groups. Feces were collected at 12, 24, 36, 48 and 60 hours after oral administration of Re (100mg/kg). Six metabolites of Re were detected in the feces of rat. The structures of the metabolites were identified as 20(S)-ginsenoside Rg_2_, 20(S)-ginsenoside Rh_1_, 20(R)-ginsenoside Rh_1_, ginsenoside F_1_, 3-oxo-ginsenoside Rh_1 _and PPT. The metabolic pathways of Re in animals were similar to those in humans [59].

A similar metabolic study was also carried out *in vivo *with HPLC coupled with electrospray ionization and quadrupole time-of-flight tandem mass spectrometry (HPLC-ESI-TOF-MS/MS) [60]. The rat urine samples were collected and pretreated through C(18) solid-phase extraction cartridges prior to analysis. As a result, eleven and nine metabolites together with Re were detected and identified in rat urine after oral and intravenous administration, respectively. Oxidation and deglycosylation were found to be the major metabolic processes of the constituent in rat, indicating that a large part of the intact ginsenosides was metabolized and transformed to ginsenosides with more biological effects in the gastrointestinal tract [52]. PPT ginsenosides, such as Re and Rg_1_, were mainly converted to Rh_1 _and F_1 _and then to corresponding aglycones [51,56].

### Elimination

Xia *et al*. [38] applied a developed and validated liquid chromatography-electrospray ionization-mass spectrometry (LC-ESI-MS) method to detect Re, Rg_1_, Rd, Rb_1 _and ophiopogonin D in rat plasma. Re and Rg_1 _were eliminated quickly from the body. The pharmacokinetic behaviors of Rd and Rb_1 _were significantly different from those of Re and Rg_1 _in rat. Joo *et al*. [27] found that Re was rapidly cleared from the body within 0.2 ± 0.03 hour for male mice and 0.5 ± 0.08 hour for female mice after intravenous administration. They also found that ginseng berry extract exhibited a superior oral absorption of Re as compared to orally fed Re, suggesting that ginseng berry extract may be of choice for Re intake [27].

The plasma concentrations of Re and Rg_1 _were determined and the pharmacokinetic parameters were calculated after intravenous *Shenmai *injection in ten volunteers [61]. The study found the distribution and elimination of Re and Rg_1 _to be rapid after intravenous injection; and the pharmacokinetic characteristics could be fitted to the two-compartment model of pharmacokinetics.

## Conclusion

Multiple biological and environmental factors affect the quantity and quality of ginsenosides in ginseng parts. Studies on Re demonstrate that (1) the absorption of Re is quick in rats; (2) PPT, Re and Rg_1_, are likely to be metabolized to Rh_1 _and F_1 _by intestinal microflora before absorption into the blood; and (3) Re can be quickly eliminated from the body.

## Abbreviations

DAS: Drug and Statistics for Windows pharmacokinetic software; ELISA: enzyme-linked immunosorbent assay; Re: ginsenoside Re (Rg_1_, Rg_2_, Rg_3_, Rf, Rb_1_, Rb_2_, Rb_3_, Rc, Rd, and Re_1 _- Re_6 _are the same abbreviations with Re); HPLC-ESI-TOF-MS/MS: high-performance liquid chromatography coupled with electrospray ionization and quadrupole time-of-flight tandem mass spectrometry; HPLC-ELSD: high-performance liquid chromatography coupled with evaporative light scattering detection; HPMAE: high pressure microwave-assisted extraction; PPD: protopanaxadiol; LC-ESI-MS: liquid chromatography-electrospray ionization-mass spectrometry; PPT: protopanaxatriol; TPNG: total *Panax *notoginsenoside; UPLC/MS: ultra performance liquid chromatography mass spectrometry

## Competing interests

The authors declare that they have no competing interests.

## Authors' contributions

JTX and LHX conceived study. DCP, HSW, CLQ and SMW collected the data. DCP, HSW, CLQ, LHX, SMW and JTX wrote the manuscript. The authors have read and approved the final version of the manuscript.
